# Anxiety in Brief: Assessment of the Five-Item Trait Scale of the State-Trait Anxiety Inventory in South Africa

**DOI:** 10.3390/ijerph20095697

**Published:** 2023-05-01

**Authors:** Tyrone B. Pretorius, Anita Padmanabhanunni

**Affiliations:** Department of Psychology, University of the Western Cape, Bellville 7530, South Africa; apadmana@uwc.ac.za

**Keywords:** STAI-T5, trait anxiety, psychometric properties, Rasch analysis, Mokken analysis, classical test theory

## Abstract

The current study examined the psychometric properties of a short form of the trait scale of the Spielberger State-Trait Anxiety Inventory. Participants consisted of a convenience sample of students (*n* = 322) who completed the five-item version of the trait scale of the State-Trait Anxiety Inventory, the Perceived Stress Scale, the nine-item version of the Beck Hopelessness Scale, the 10-item version of the Center for Epidemiological Studies Depression Scale, and the Post-Traumatic Stress Disorder Checklist. We used classical test theory and item response theory (Rasch and Mokken analyses) to examine the psychometric properties of a previously proposed five-item version of this scale. These approaches confirmed that the five-item measure of anxiety had satisfactory reliability and validity, and also confirmed that the five items comprised a unidimensional scale.

## 1. Introduction

Anxiety is an adaptive emotional response and is typically characterized by an intense sense of apprehension, tension, and worry. It is conceptualized as a future-oriented mood state associated with preparation for impending adverse events or stressors. Symptoms of anxiety include worry (i.e., internal and subjective responses), withdrawal or avoidance (i.e., overt behavioral acts), and physiological reactions (e.g., heart palpitations or muscle tension). Although anxiety can facilitate coping, if one has excessive and unmotivated anxiety, that anxiety can become dysfunctional and lead to the development of an anxiety disorder (e.g., generalized anxiety disorder or social anxiety disorder). Anxiety can be differentiated from fear in that the latter represents an alarm response to present or imminent danger [1].

Theoretical conceptualizations of anxiety can be traced back to Freud [2], who proposed three forms of anxiety—namely, objective anxiety, which is based on actual physical threats, and neurotic and moral anxiety, both of which are produced by intrapsychic conflicts (e.g., between the id and superego). Subsequent conceptualizations of the cognitive behavior of anxiety have underscored the role of maladaptive appraisals in influencing emotional responses [3]. According to this perspective, heightened negative or distorted appraisals of situations or stressors can produce negative emotional reactions such as anxiety. The use of dysfunctional coping strategies such as withdrawal and avoidance can further maintain anxiety levels and lead to psychopathology [3].

One of the central debates in the field of anxiety research has involved the distinction between trait and strait anxiety [4,5]. Trait anxiety is considered a personality feature or characteristic predisposition to appraise events or stressors as potentially threatening, thereby resulting in anxiety [5]. It is considered to be a vulnerability factor for the development of mental health problems. State anxiety, in contrast, refers to the transitory experience of the emotion of anxiety in response to perceived stressors. There has also been debate as to whether anxiety is a unidimensional or a multidimensional construct. According to Spielberger’s [6] seminal state-trait anxiety theory, anxiety is a unidimensional construct. External stressors and internal experiences (e.g., physiological responses) are appraised in a way that can potentially lead to anxiety, and the appraisal process is influenced by trait anxiety (i.e., personality-related predisposition toward anxiety). In other words, general trait anxiety predisposes an individual to increases in state anxiety in situations appraised as threatening. Other researchers (e.g., [7]) have proposed that both state and trait anxiety are multidimensional in nature. State anxiety has been conceptualized as consisting of two basic components—namely, cognitive appraisals and autonomic or emotional reactions—while trait anxiety is viewed as comprising four facets associated with specific situations or stressors—namely, social evaluation threats, physical danger threats, ambiguous threats, and threats related to innocuous situations [8]. Spielberger’s [6] State-Trait Anxiety Inventory (STAI) remains the most long-standing and commonly used self-report measure for anxiety; as a result, most of the research on anxiety assessment relies on a unidimensional approach.

Over the past decades, numerous scales have been developed to measure trait anxiety. Elwood and colleagues [4] provided a comprehensive review and synthesis of the eight most frequently used measures of trait anxiety—namely, the 21-item Beck Anxiety Inventory, the 40-item State-Trait Anxiety Inventory (STAI), the 14-item Cognitive-Somatic Anxiety Questionnaire, the 60-item Endler Multidimensional Anxiety Scale-Trait, the seven-item Hospital Anxiety and Depression Scale, the 60-item Four Systems Anxiety Questionnaire, the 36-item Three Systems Anxiety Questionnaire, and the 42-item State-Trait Inventory for Cognitive and Somatic Anxiety.

The COVID-19 pandemic has further propelled the development of anxiety scales, including the COVID-19 Anxiety Scale (CAS: [9]), the Coronavirus Pandemic Anxiety Scale (CPAS-11: [10]), and the New COVID-19 Anxiety Scale (NCAS) [11]. The CAS is a 5-item measure of COVID-19-related anxiety and is a unidimensional tool. The shortcomings of the instrument have been highlighted by several authors (e.g., [10]) who argue that the CAS measures symptoms of generalized anxiety and may not adequately capture anxiety related to the pandemic. Bernado and colleagues [10] developed the 11-item CPAS, but the instrument has been criticized [11] owing to the items being generic and the possibility of the measure not tapping into anxiety specifically related to the disease outbreak. The NCAS was developed to address these shortcomings and comprises 12 items. The developers [11] provide evidence of the psychometric properties of the instrument when used in India. In sum, the instruments developed to investigate anxiety related to the COVID-19 pandemic appear to measure state anxiety and, given the recency of their development, require further psychometric testing in different contexts and using different samples.

The STAI [6] was developed to provide a reliable and relatively brief self-report measure to assess both state and trait anxiety. The original instrument consisted of two 20-item subscales that measured the intensity of anxiety that a person experiences in the moment (STAI-State or STAI-S subscale) and the frequency with which an individual “generally feels” anxious (STAI-Trait or STAI-T subscale). While the first STAI questionnaire was developed in 1970 (Form X), the second was formulated in 1983 (Form Y) and replaced six of the 20 items to improve the scale’s factor structure and its ability to discriminate between anxiety and depression [6].

The STAI has been used in numerous studies and has been translated into over 70 languages [12]. It has been lauded for being a brief, inexpensive, easy-to-administer, and thorough measure of trait anxiety. The STAI-T has demonstrated sound internal consistency reliability and construct validity [12] and is able to discriminate between psychiatric patients and healthy control subjects, making it effective in measuring changes in anxiety over time. It has also been successfully correlated with other measures of anxiety [13]. Nevertheless, some of its psychometric properties have been criticized. While the STAI-T has demonstrated sound test-retest reliability in normative samples, various studies have reported that trait anxiety scores fluctuated when measured in different situations and diverse population groups (e.g., [12,14]), suggesting poor reliability. The STAI has also been criticized for poor discriminant validity due to its strong correlation with measures of depression compared to anxiety [12]. Notably, a few studies using the STAI-T have examined its psychometric properties or examined measurement invariance [15].

One of the notable concerns with the STAI relates to its length. The instrument can prove to be time-consuming, particularly in surveys assessing more than one variable [15]. Furthermore, lengthy questionnaires that assess anxiety may inadvertently produce test anxiety during its completion, potentially inflating overall anxiety scores and impacting the validity of findings. Respondents have been found to lose interest when completing long questionnaires, leading to the elicitation of unthoughtful responses or nonresponse and thereby impacting the results [16]. A shorter-form STAI could thus mitigate this issue and produce more reliable results. Short forms of scales tend to be relatively brief to administer and do not require costly or time-consuming scoring or interpretation procedures [16]. For these reasons, there has been a growing need to develop short measures with sound psychometric properties. There have been various attempts at creating a short and valid version of the STAI (e.g., [17,18]), but these attempts have suffered from various methodological shortcomings, including the use of inappropriate samples and the inclusion of reverse-scored items. To address these shortcomings, Zsido and colleagues [5] developed the short five-item version of the STAI-T by firstly removing all the reverse-scored items, and secondly using item-response theory to identify items that discriminated the best among participants. The current study examines the psychometric properties of the five-item short version of the trait scale of the STAI (STAI-T5) using both classical test theory (CTT) and item response theory (IRT)—specifically, Rasch and Mokken analyses. It should be noted that the STAI assesses both state (i.e., anxiety in relation to a specific situation) and trait (i.e., a personality trait that predisposes and individual to experiencing state anxiety in adverse situations). For our purpose, we only focused on trait anxiety as a general way of seeing the world.

The psychometric properties of instruments are most frequently examined using CTT. However, combining these different approaches provides a more holistic view of an instrument since each of these approaches provides different yet complementary information on the function of an instrument. CTT provides scale-level indices, such as reliability, whereas IRT focuses more on item-level indices such as item difficulty [19]. It has also been empirically demonstrated that IRT indices are less sample-dependent than CTT indices [20]. While CTT indices can vary greatly across samples, IRT indices are relatively more stable across different samples. Mokken analysis is a non-parametric alternative to Rasch analysis, thus it contains fewer assumptions than Rasch analysis.

The goal of our study is to provide a more comprehensive picture of the psychometric properties of the instrument under investigation through the use of CTT and IRT indices. By combining these approaches, we aimed to generate additional information on the functioning of the instrument when used in a diverse context. Furthermore, we were interested in determining the sensitivity of the short-form of the STAI-T in detecting differences between groups based on their COVID-19-related experiences. Fear and anxiety were dominant emotional responses to the COVID-19 pandemic, but differences in the levels of anxiety based on COVID-19-related experiences (e.g., increased risk of contagion due to occupation, having elderly family members, etc.) have been documented in the literature (e.g., [21]). We were therefore interested in the sensitivity of the STAI-T to these differences.

## 2. Materials and Methods

### 2.1. Participants

Participants consisted of a random sample of students (*n* = 322) at a university in the Western Cape Province of South Africa. This sample size contained a 5.13% margin of error (95% confidence interval). A description of the sample is presented in Table 1. The majority of the sample were women (77%) who lived in an urban area (87.3%). The mean age of the sample was 26 years (*SD* = 10.2). We also collected data about participants’ COVID-19-related experiences as we wanted to determine whether the short form of the STAI-T would be sensitive enough to detect differences between groups depending on their COVID-19 experiences. The majority of participants (80.1%) knew people who had COVID-19, while 25.5% had tested positive for COVID-19, 86.6% of the sample were vaccinated, and 40.7% had lost a family member due to COVID-19.

### 2.2. Instruments

Participants completed the following instruments: the five-item version of the trait scale of the State-Trait Anxiety Inventory (STAI-T5) [5], the Perceived Stress Scale (PSS) [22], the nine-item version of the Beck Hopelessness Scale (BHS-9) [23], the 10-item version of the Center for Epidemiological Studies Depression Scale (CES-D10) [24], and the Post-Traumatic Stress Disorder Checklist (PCL-5) [25]. In addition, participants completed a brief demographic questionnaire consisting of the items listed in Table 1.

The STAI-T5 is a short form of the original 20-item trait scale of the State-Trait Anxiety Inventory (STAI-T) [26], and consists of five items assessing trait anxiety. It is scored on a 4-point scale ranging from “not at all” (1) to “very much so” (4). An example item of the STAI-T5 is: “I get in a state of tension or turmoil as I think over my recent concerns and interests.” Zsido and colleagues [5] reported sound psychometric properties for the shortened version of the STAI-T that were comparable to the properties of the longer form. In particular, they reported a reliability coefficient of 0.86 for the short form, and the correlation between the STAI-T5 and other indices of psychological well-being served as evidence for the validity of the short form.

The PSS is a 10-item scale that measures generalized perceptions of stress. Participants respond to the items using a 5-point scale that ranges from “never” (0) to “very often” (4). A sample item of the PSS is: “How often have you been upset because of something that happened unexpectedly?” Cohen [22] reported a reliability coefficient of 0.78 and confirmed criterion-related validity through relationships with other measures of stress appraisal.

The BHS-9 is a shortened version of the original Beck Hopelessness Scale [26]. The response format is dichotomous—namely, “true” (1) or “false” (0). An example item from the BHS-9 is: “I might as well give up because there is nothing I can do about making things better for myself.” Balsamo and colleagues [23] reported an alpha coefficient of 0.86 for the nine-item BHS and also demonstrated that the short form was able to discriminate between psychiatric inpatients at high risk of suicide versus those at low risk of suicide.

The CES-D10 is a short form of the original 20-item version of the CES-D [27], and participants respond to the 10 items on a 4-point scale that ranges from “most or all of the time” (3) to “rarely or none of the time” (0). An example item from the CES-D10 is: “I was bothered by things that usually do not bother me.” Zhang and colleagues [24] reported a reliability coefficient of 0.88 and demonstrated that the short version was as accurate as the 20-item version in classifying participants with depressive symptoms.

The PCL-5 is a 20-item measure of the presence and severity of PTSD symptoms. Participants respond to the items of the PCL-5 using a 5-point scale that ranges from “not at all” (0) to “extremely” (4). An example of the items from the PCL-5 is: “How much have you been bothered by trouble remembering important parts of the stressful experience?” Blevins and colleagues [25] reported reliability coefficients of 0.94 and 0.95 in two separate studies with trauma-exposed students, and also provided evidence of convergent and discriminant validity.

### 2.3. Procedure

We created an electronic version of the instruments using Google Forms. The Office of the Registrar distributed the link to a random sample of 1500 students. We thus had a response rate of 21.5%. The link stayed active for a period of three months (May–July 2022), and reminders were sent twice per month to the selected students. The instrument was only available in English as this is the medium of instruction at the University level.

### 2.4. Ethics

The study was conducted according to the guidelines of the Declaration of Helsinki and was approved by the Humanities and Social Sciences Ethics Committee of the University of the Western Cape (ethics reference number: HS21/5/23). Participants provided informed consent, participation was voluntary and anonymous, and no incentives were provided for participation.

### 2.5. Data Analysis

IBM SPSS for Windows Version 28 (IBM Corp., Armonk, NY, USA) was used to obtain the following CTT indices: reliability (Cronbach’s alpha), composite reliability (CR), average variance extracted (AVE), maximum shared variance (MSV), average shared variance (ASV), item-total correlations, inter-item correlations, standard error of measurement, and exploratory factor analysis (EFA: principal components). AVE refers to the amount of variance in the items accounted for by the latent construct; MSV refers to the amount of variance that the latent construct has in common with the variable most strongly correlated with it; and ASV refers to the average amount of variance that the latent construct has in common with all variables that it is correlated with. SPSS was also used to obtain the intercorrelations (Pearson’s *r*) between anxiety and variables included for the purpose of examining criterion-related validity: perceived stress, hopelessness, depression, and PTSD. In addition, confirmatory factor analysis (CFA) was conducted using IBM SPSS AMOS for Windows Version 27 (IBM Corp.). The fit indices that were used to examine model fit included chi-squared (χ^2^), the goodness-of-fit index (GFI), the comparative fit index (CFI), the Tucker-Lewis index (TLI), and the standardized root mean squared residual (SRMR), as suggested by Kline [28].

Rasch indices were obtained using Winsteps (Version 5.1.4) [29], and included item and person separation indices, item and person reliability, and the infit and outfit mean square (*MnSq*). Item and person indices provide an indication of whether items are able to differentiate between participants with high and low levels of anxiety (person separation indices) and whether an item-difficulty hierarchy is present (item separation indices) [29]. The *MnSq* statistics evaluate the extent to which each item fits the Rasch dimension. In addition, a principal components analysis (PCA) of the residuals in Rasch (after the presumed latent variable is accounted for) was conducted to determine whether there are additional dimensions over and above the Rasch dimension.

Mokken indices were obtained with the “Mokken” package [30] in R [31], and included Mokken scale reliability coefficients (*MS_rho_*), scalability coefficients for individual items (*H_i_*), scalability coefficients for the total scale (*H*), and *Crit* values for monotonicity and invariant item ordering (IIO). The assumption of monotonicity refers to the extent to which an item is able to effectively distinguish between participants with high and low levels of anxiety. IIO provides an indication of whether there are items that respondents with the same level of anxiety might have endorsed in significantly different ways [32]. The *Crit* value provides a statistical indication of whether the assumptions of monotonicity and IIO have been violated.

The reliability of the STAI-T5 was thus evaluated with Cronbach’s alpha (*α*), *CR* and *MS_rho_*. Inter-item correlations, item-total correlations, and factor loadings in CFA (CTT), H_i_ for individual items (Mokken), item and person indices, as well as infit and outfit *MnSq* (Rasch) were used to evaluate the construct validity of the STAI-T5. AVE, CR, and factor loadings were used to evaluate convergent validity. The discriminant validity of the STAI-T5 was examined by comparing AVE, MSV, and ASV, as one would expect that the latent construct should have more in common with the items that contribute to its measurement (AVE) than with other related constructs (MSV and ASV) [33]. Criterion-related validity was evaluated in terms of the relationship between anxiety and related constructs such as perceived stress, hopelessness, depression, and PTSD.

The dimensionality of the STAI-T5 was examined with EFA and CFA in CTT, the scalability coefficient for the scale (*H*) in Mokken analysis, and a PCA of the residuals in Rasch analysis. In addition, Mokken analysis uses an algorithm called the automated item selection procedure (AISP), which partitions items into scales and indicates whether more than one dimension underlies the items. To coherently present and interpret the findings, the acceptable level for all the CTT, Rasch and Mokken indices, and the appropriate cutoff points and decision rules were integrated with the obtained results.

Lastly, we examined differences in anxiety between various groups (those who had lost family due to COVID-19 and those who did not; those that tested positive for COVID-19 and those who did not; and those who were vaccinated and those who were not) using a *t*-test.

## 3. Results

The descriptive statistics for, the intercorrelations between, and the reliabilities of the study’s variables are reported in Table 2. The *α* and *ω* coefficients in Table 2 all exceed the conventional cutoff for acceptable reliability (>0.70) [34] and ranged between 0.84 and 0.94. Anxiety was significantly associated, in the expected direction (positive), with perceived stress (*r*(320) = 0.60, *p* < 0.001), hopelessness (*r*(320) = 0.46, *p* < 0.001), depression (*r*(320) = 0.66, *p* < 0.001), and PTSD (*r*(320) = 0.66, *p* < 0.001), providing evidence for criterion-related validity.

The CTT, Rasch, and Mokken indices for the items of the STAI-T5 are presented in Table 3. In this table, the inter-item correlations are reported below the diagonal. Paulsen and BrckaLorenz [35] suggest that inter-item correlations should ideally be between 0.15 and 0.85, that inter-item correlations greater than 0.85 indicate redundancy of items, and that coefficients lower than 0.15 indicate that items do not have much in common. As can be seen in Table 3, the items of the STAI-T5 demonstrated a high degree of commonality, as inter-item correlations ranged between 0.53 and 0.85, and none were greater than 0.85.

In general, significant factor loadings and loadings above 0.70 also support the convergent validity of an instrument [36,37]. All the factor loadings in Table 3 are both significant and above 0.70. The construct validity of an instrument is also supported if the item-total correlations are significant and >0.50, as it demonstrates that all items contribute to the measurement of the latent variable [38,39]. In Table 3, the item-total correlations ranged between 0.64 and 0.75, and all were significant. As indicated, the *MnSq* statistics indicate the extent to which the items fit the Rasch model and provide evidence for construct validity. Linacre [29] suggested that infit and outfit *MnSq* values < 0.50 and >1.50 indicate misfitting items. None of the *MnSq* values were below 0.50 or above 1.50, and ranged between 0.84 and 1.16. The scalability coefficient of the individual items (*H_i_*) serves the same function as the item-total correlations in CTT, as it indicates the extent to which all items contribute to the measurement of the latent variable. Mokken [40] proposed that *H_i_* coefficients > 0.30 are indicative of items that contribute to the measurement of the latent construct. With regard to monotonicity and IIO, it has been suggested that *Crit* values > 80 indicate serious violations of these two assumptions [32]. As can be seen in Table 3, only Items 4 and 5 were flagged as potentially violating IIO, but the associated *Crit* values for these two items (19) were far below 80. There were no violations of monotonicity.

**Figure 1:**
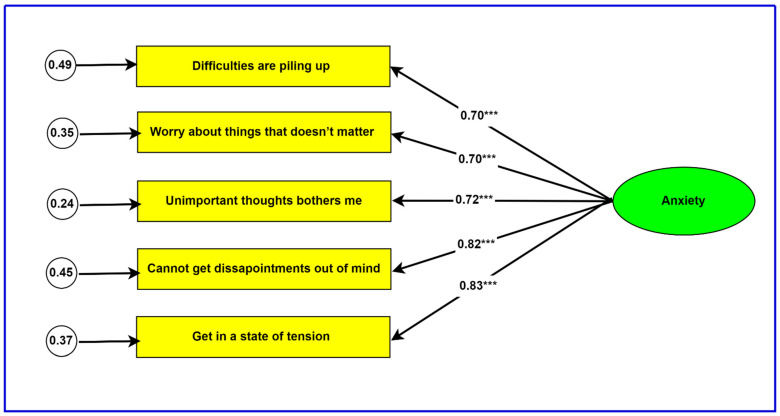
Confirmatory factor model of the STAI-T5. Note: rectangles are observed variables, ellipse is a latent variable and circles are error variances. All error variances are significant. *** *p* < 0.001.

To examine the dimensionality of the STAI-T5, EFA (principal components analysis) and CFA were conducted. The EFA extracted one factor that accounted for 67.7% of the variance. The factor loadings associated with the one-factor model are reported in Table 3. The one-factor model of the STAI-T5 that was evaluated with CFA is presented in Figure 1, together with associated factor loadings.

The fit indices associated with the one-factor model in Figure 1 were: *χ*^2^ = 27.3, *p* < 0.001; *GFI* = 0.97; *CFI* = 0.97; *TLI* = 0.91; *SRMR* = 0.04. The literature has suggested the following best fit indicators: *χ*^2^ = non-significant; *GFI* > 0.95; CFI > 0.90; TLI > 0.90; SRMR < 0.08 [41]. With the exception of *χ*^2^, the fit indices obtained in the current study provide support for a one-factor model of the STAI-T5. However, it has been indicated that the *χ*^2^ is a test of perfect fit and is affected by sample size [42].

The CTT, Rasch and Mokken indices at the scale level are reported in Table 4. As reflected in Table 4, all the indices of reliability (*α*, *CR* and *MS_rho_*) may be considered satisfactory, as they were >0.70. Posch [36] indicated that an AVE > 0.50 and less than CR provides evidence of convergent validity. In Table 4, AVE was 0.69 and also less than CR (0.92). An AVE greater than MSV and ASV provides evidence of discriminant validity [33]; thus, the AVE of 0.69, the MSV of 0.44, and the ASV of 0.36 support the discriminant validity of the STAI-T5. Linacre [29] proposed that a person separation index of >2, as well as a person reliability of >0.80, would indicate that the instrument can distinguish between high and low scorers. In Table 4, the person separation index of 2.19 and the person reliability of 0.83 provide evidence that the items of the STAI-T5 can differentiate between those with high and low levels of anxiety. In contrast, an item separation index > 3 and item reliability > 0.80 confirm that an item-difficulty hierarchy exists. As can be seen in Table 4, the item separation index of 4.82 and the item reliability of 0.96 confirm the item-difficulty hierarchy of the STAI-T5. With regard to dimensionality, Linacre proposed that if an eigenvalue of a possible second dimension extracted through a PCA of the residuals in Rasch is >2, the instruments are multidimensional [29]. In this instance, the Rasch analysis confirmed the results of the EFA and CFA, as the eigenvalue of the second dimension extracted with a PCA of the residuals was 1.93. AISP in Mokken analysis also confirmed that all the items of the STAI-T5 loaded on a single factor. With respect to the *H* coefficient, Wind [43] suggested that an H coefficient > 0.50 indicates the existence of a very strong scale. In the current study, *H* was 0.64, indicating that the STAI-T5 represents a very strong scale.

Lastly, the results of *t*-test analyses indicated that participants who had tested positive for COVID-19 (mean = 12.74, *SD* = 4.00) did not differ significantly from those who had tested negatively (mean = 12.01, *SD* = 4.14, t = 1.35, *p* > 0.05). However, participants who had been vaccinated (mean = 12.64, *SD* = 4.04) reported higher levels of anxiety than participants who had not been vaccinated (mean = 10.52, *SD* = 4.33, t = 3.20, *p* = 0.002). Moreover, participants who lost a family member due to COVID-19 (mean = 12.95, *SD* = 4.11) reported higher levels of anxiety than those who had not lost a family member (mean = 11.96, *SD* = 4.11, t = 2.12, *p* = 0.035).

## 4. Discussion

The current study examined the psychometric properties of a five-item version of the trait scale of the State-Trait Anxiety Inventory from the perspectives of CTT and item response theory—more specifically, Mokken and Rasch analyses. All three approaches provided evidence for the reliability, validity, and unidimensionality of the STAI-T5. These results confirm those obtained by Zsido and colleagues [5], who used the graded response model to examine the psychometric properties of each item.

The instrument demonstrated satisfactory reliability in terms of Cronbach’s alpha, CR, and Mokken scale reliability. In terms of construct validity, inter-item correlations were not too low or too high, demonstrating that there were not redundant items, nor did the items have much in common. The item-total correlations were all significant, providing further evidence of construct validity, as these item-total correlations demonstrated that all items contribute to the measurement of the latent construct. This was also confirmed by the scalability coefficients of the individual items obtained in the Mokken analysis. Furthermore, *MnSq* values were within an acceptable range, and both the person indices in the Rasch analysis and the monotonicity in the Mokken analysis confirmed that the five items of the STAI-T5 are able to distinguish between participants with high levels of anxiety and those with low levels of anxiety. IIO in the Mokken analysis revealed that there were no items that respondents with the same level of anxiety might have responded to in significantly different ways. The item separation indices in the Rasch analysis also indicated the existence of an item-difficulty hierarchy.

With regard to convergent validity, AVE was greater than 0.50 and less than CR. This demonstrates that the variance explained by the latent construct is greater than measurement error or cross-loadings [33]. In terms of discriminant validity, the latent construct of anxiety explained more of the variance in the items that contributed to its measurement (AVE) than it had in common with other related variables (MSV, ASV). Anxiety also had significant associations with perceived stress, hopelessness, depression, and PTSD, thus providing evidence for criterion-related validity.

AISP in the Mokken analysis indicated that the five items loaded on a single scale. A PCA of the residuals in the Rasch analysis indicated that a possible second dimension had a low eigenvalue and that one dimension was therefore sufficient to account for the variance in the items. EFA extracted one factor, and CFA confirmed that a one-factor model is a good fit for the data. Taken together, these results provide strong evidence for the unidimensionality of the STAI-T5.

The current study has important implications. It lends support to the reliability and validity of the STAI-T5 and underscores that trait anxiety is a clinically meaningful construct to measure. The STAI-T’s brevity and ease of scoring make it an ideal screening tool in the context of clinical assessment. Scores upon intake and follow-up could potentially be used as part of a risk profile assessment and facilitate targeted interventions for those with high levels of anxiety. The scale’s ability to detect differences between groups depending on their COVID-19-related experiences underscores the sensitivity of the instrument and its potential utility for identifying those in need of differentiated interventions.

The study had several limitations. First, a cross-sectional survey design was used, limiting the extent to which causal inferences can be drawn; longitudinal research is thus recommended to confirm the findings of the study. Second, the sample was disproportionately female and consisted predominantly of primary school teachers; more balanced samples are needed to lend support to the findings. Third, various unmeasured variables could have impacted the results (e.g., prior history of mental illness, exposure to trauma, etc.). Further information on the role of these factors on the scores of the STAI-T5 is needed. Fourth, the study relied on self-report measures and was therefore susceptible to respondent and social desirability biases. Finally, since trait anxiety refers to an individual’s anxiety proneness, it is probable that those with a predisposition to anxiety may experience greater symptoms of anxiety in response to COVID-19-related stressors and score higher on the instrument. This may have a bearing on the results.

## 5. Conclusions

The current study used a rigorous psychometric approach to assess the psychometric properties of the STAI-T5, and concluded that the instrument appears to be a valid and reliable measure of anxiety. By using both Rasch and Mokken analyses, our study complements previous studies that have mostly used CTT. We recommend that future research assess the psychometric properties of the instrument with larger and more diverse samples. Additional evidence of the psychometric strengths of the STAI-T5 may support its use in routine clinical practice and in public health care settings.

## Figures and Tables

**Table 1:** Description of the sample.

Variable	Categories	*n*	%
Gender	Male	68	21.1
	Female	248	77
	Non-Binary	4	1.2
	Transgender	2	0.6
Area of residence	Rural	41	12.7
	Urban	281	87.3
Do you know people infected	Yes, confirmed	258	80.1
	Suspected but not confirmed	21	6.5
	Negative	8	2.5
	No	35	10.8
Tested positive for COVID-19?	Yes	82	25.5
	No	177	55
	Suspected	63	19.6
Received a vaccination?	Yes	279	86.6
	No	43	13.4
Lost family	Yes	131	40.7
	No	191	59.3
Mean age ± *SD*		26 ± 10.2	

**Table 2:** Descriptive statistics for, reliabilities of, and intercorrelations between study variables.

	1	2	3	4	5
Anxiety	--				
Stress	0.60 **	--			
Hopeless	0.46 **	0.47 **	--		
Depression	0.66 **	0.66 **	0.50 **	--	
PTSD	0.66 **	0.67 **	0.47 **	0.68 **	--
Mean	12.3	23.9	2.3	14.1	38.5
*SD*	4.1	6.3	2.4	6.8	18.9
Alpha	0.88	0.85	0.84	0.84	0.94
Omega	0.88	0.86	0.84	0.85	0.94

** *p* < 0.001.

**Table 3:** Indices for the items of the STAI-T5.

Item	1	2	3	4	5
1. Difficulties are piling up	--				
2. Worry about things that do not matter	0.53 ***	--			
3. Unimportant thoughts bothers me	0.54 ***	0.78 ***	--		
4. Cannot get disappointments out of mind	0.53 ***	0.57 ***	0.61 ***	--	
5. Get in a state of tension	0.59 ***	0.57 ***	0.56 ***	0.69 ***	--
Factor loadings	0.77 ***	0.84 ***	0.85 ***	0.83 ***	0.83 ***
Item-total correlations	0.64 ***	0.73 ***	0.75 ***	0.72 ***	0.73 ***
Infit *MnSq* (Rasch)	1.14	0.96	0.84	1.05	0.97
Outfit *MnSq* (Rasch)	1.16	0.95	0.85	1.02	0.98
*H_i_* (Mokken) ^a^	0.62	0.65	0.67	0.63	0.64
SE of *H* (Mokken)	0.04	0.03	0.03	0.03	0.04
*Crit* value for monotonicity (Mokken)	0	0	0	0	0
*Crit* value for IIO (Mokken)	0	0	0	19	19

Note. Item intercorrelations below the diagonal. ^a^ Scalability coefficient of individual items. *** *p* < 0.001.

**Table 4:** Classical test theory, as well as Rasch and Mokken indices for the STAI-T5 at the scale level.

Index	Value	Suggested Cutoff
Cronbach’s alpha (*α*)	0.88	>0.7
Composite reliability (*CR*)	0.92	>0.7
Average variance extracted (*AVE*)	0.69	>0.5
Maximum shared variance (*MSV*)	0.44	<AVE
Average shared variance (ASV)	0.36	<AVE
Standard error of measurement	2.12	Small values
Item separation reliability (Rasch)	0.96	>0.8
Item separation index (Rasch)	4.82	>3
Person separation reliability (Rasch)	0.83	>0.8
Person separation index (Rasch)	2.19	>2
Unexplained variance in the first contrast (Rasch)	1.93 ^a^	<2
Scale *H* (Mokken)	0.64	>0.50
Mokken scale reliability (*MS_rho_*)	0.89	>0.70

Note. ^a^ Eigenvalue.

## Data Availability

The raw data supporting the conclusions of this article will be made available by the authors, without undue reservation.
